# Translation and Cross-cultural Adaptation of the SOSG-OQ 2.0 Questionnaire into Brazilian Portuguese *

**DOI:** 10.1055/s-0043-1775890

**Published:** 2024-03-21

**Authors:** Matheus Batista, Gabriel Pokorny, Carlos Augusto Belchior Bitencourt Júnior, Marcella de Almeida Bento, Thabata Pasquini Soeira, Carlos Fernando Pereira da Silva Herrero

**Affiliations:** 1 Instituto de Patologia da Coluna, São Paulo, SP, Brasil; 2 Instituto de Assistência Médica ao Servidor Público Estadual (Iampse), São Paulo, SP, Brasil; 3 Faculdade de Medicina de Ribeirão Preto, Universidade de São Paulo, Ribeirão Preto, SP, Brasil; 4 Centro Universitário Estácio de Ribeirão Preto, Ribeirão Preto, SP, Brasil

**Keywords:** spine/surgery, surveys and questionnaires, neoplasm metastasis, quality of life, translations

## Abstract

**Objective:** To perform the cross-cultural adaptation and translation into Brazilian Portuguese of the Spine Oncology Study Group – Outcomes Questionnaire 2.0 (SOSG-OQ 2.0) to enable its application to Brazilian patients and to allow Brazilian researchers to use a questionnaire that is on trend in the scientific literature.

**Materials and Methods:** The present is a basic, non-randomized, non-comparative study. The translation followed the proposal by Reichenheime and Moraes, mainly for the semantic equivalence and measurement equivalence sessions, as well as the recommendations by Coster and Mancini mainly in the translation stage. The stages were as follows: first – translation into Brazilian Portuguese; second – back-translation; third – semantic comparison; fourth – validation of the final construct.

**Results:** The translations of the SOSG-OQ 2.0 made by three translators presented a high degree of similarity for most questions. The translators kept all question titles and subtitles, as well as their internal and external orders. Two sworn translators, with native proficiency in English, performed the back-translation of the amalgamated text. Both back-translations were quite similar, and any differences were solved through consensus between the main author and the sworn translators, and the translated text was considered the final version.

**Conclusion:** The present study shows a translated version of the SOSG-OQ 2.0 with semantic validity with the original version published in English. As such, researchers can apply the questionnaire to the Brazilian population, adding another tool for spine surgeons to improve the monitoring of this complex group of patients.

## Introduction

In Brazil, since 2000, cancer has been the second leading cause of death after heart disease. 1 The prevalence of metastatic spinal tumors is higher than that of primary tumors at this location. 2 3 Metastatic spinal disease increases the morbidity related to the primary condition, directly impacting the patient's quality of life. 2 4 5

It is not uncommon for patients with metastatic disease to present with dysfunctions in several body systems, and they may undergo different treatments, including chemotherapy and/or radiotherapy. 6 Sometimes, these subjects require spinal surgery to preserve or restore neurological function, sustain spinal segmental stability, and control pain. 6 7 8

Multiple tools are currently available to study the clinical outcomes of patients with metastatic spinal tumors. However, they are nonspecific and usually analyze a single variable. 9 For instance, the Frankel scale and the American Spinal Injury Association (ASIA) impairment scale quantify (classify) the degree of neurological injury. 10 11 In addition, quality-of-life questionnaires filled out by patients determine how they perceive their quality of life/health status, enabling them to identify the impact of a procedure or condition on the subject quality of life/health status. 12 13 In spinal surgery, the most applied quality-of-life questionnaires are the Oswestry Disability Index 14 and the Neck Disability Index, 15 which specifically quantify the impact of a condition on the self-perceived quality of life regarding the lumbar spine and cervical spine respectively. Moreover, broader questionnaires, such as the EuroQoL Five Dimensions (EQ-5D) or the 36-Item Short Form Survey (SF-36), quantify quality of life more comprehensively, without focusing on a specific condition or location, enabling the comparison of patients with different diseases or treatments using the same score. 16 17 Lastly, quality-of-life impact scores filled out by physicians, surgeons, or both, such as the Eastern Cooperative Oncology Group (ECOG) score, classify how much the tumor impacts the patient's activities. On the ECOG, 0 equals regular quality of life, while 5 indicates death. 18

Despite being validated and helpful in the follow-up and evaluation of patients with metastatic tumors, none of these questionnaires focus specifically on patients with spinal tumors. As such, the literature diverges on the best combination of questionnaires to follow-up patients with metastatic spinal tumors. For instance, Street et al. 9 recommend ECOG and SF-36, while Choi et al. 19 prefer the EQ-5D.

The lack of questionnaires for the specific evaluation of a given condition led the Spine Oncologic Study Group (SOSG) to develop an outcomes questionnaire (SOSG – Outcomes Questionnaire, SOG-OQ) to assess the quality of life of patients with metastatic spinal tumors. 20 Furthermore, a study 21 showed that the SOSG-OQ was superior to the 3-Level Version of the EQ-5D (EQ-5D-3L) in patients with metastasis, lymphoma, or myeloma. Moreover, the SOSG-OQ was more effective than the Patient-Reported Outcomes Measurement Information System (PROMIS) 22 in analyzing the quality of life of patients with spinal metastasis; however, the PROMIS was more effective in assessing physical function and pain, according to a study by Paulino Pereira et al. 23

Although specifically designed for patients with spinal metastases, the SOSG-OQ still contained certain internal inconsistencies, and items in selected subdomains did not correlate as effectively. 21 24

Thus, in 2018, Veersteg et al. 24 performed a psychometric study on the SOSG-OQ, and developed an updated version. To solve discrepancies in the first version of the SOSG-OQ , the authors divided the original question 8 (on bowel and bladder function) into 2 separate questions to facilitate the answer, since often only the bowel or bladder is dysfunctional. Furthermore, they moved questions 7 (on walking assistance) and 20 (on leaving the house) to the physical function domain; question 16 was moved to the pain domain, and question 15 (on energy level) was removed, as it was not associated with any domain and did not provide enough significant information. 24 Then, the SOSG-OQ 2.0 construct was compared with the Numeric Rating Scale (NRS) for pain and the SF-36 in patients with spinal metastasis, and a strong correlation was found between the questionnaires. 24

As such, the present study aimed to perform the cross-cultural adaptation and translation of the SOSG-OQ 2.0 into Brazilian Portuguese, to enable its application to Brazilian patients.

## Materials and Methods

The present is a basic, non-randomized, non-comparative study.

### Translation and cross-cultural adaptation process

The translation and cross-cultural adaptation of an instrument involve multiple steps to ensure that the translated construct is valid and equivalent, and that it also makes sense for the target audience. 25 The process begins with multiple translations of the original questionnaire; then, a synthesis of these texts forms the amalgamated translation. After a consensus on the translation, a group of translators (with native proficiency in the original language) performs back-translations (BTs) of the document, which are then synthesized to obtain the final BT. An expert committee compares the BT with the original version to check for any discrepancies between the previous texts. If there are few or no discrepancies, the construct undergoes psychometric and validity assessments in the target population. 25 26

**Fig. 1:**
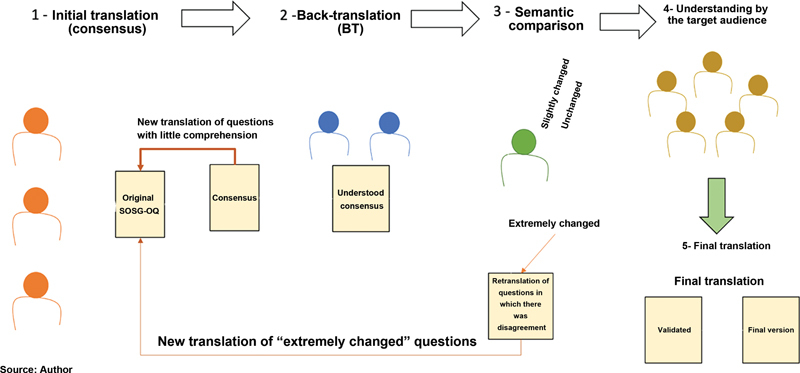
Flowchart of the translation of the SOSG-OQ until the final version.

The translation followed the proposal by Reichenheime and Moraes, 26 mainly for the semantic equivalence and measurement equivalence sessions, and the recommendations by Coster and Mancini, 27 mainly in the translation stage ( Fig. 1 ).

**Step 1:** individual translation into Brazilian Portuguese of the SOSG-OQ 2.0 by 3 Brazilian researchers. Through a comparison of the three translations a consensual, unified version was developed, called amalgamated translation. During the translation stage, the reviewers were asked to make any required changes to elements of the questions that were not so familiar to the Brazilian population.

**Step 2:** Two proofreaders, certified language studies specialists with proficiency in English, analyzed the amalgamated translation. The generated texts were called amalgamated BT.

**Steps 3 and 4:** Another translator (called final translator), who had not been involved in any of the translations and BTs, compared the amalgamated BT with the original version to provide an opinion on the similarity of the questionnaires, both in denotative and connotative aspects. This translator evaluated the questions as *unchanged* , *slightly changed* , and *extremely changed* .

### SOSG-OQ 2.0

The SOSG-OQ 2.0 was developed in 2018 as an adaptation of the original SOSG-OQ to improve the internal validity of its domains and its correlation with other previously-validated constructs. 24 The reliability values of the questionnaire in the test-retest evaluations ranged from 0.58 to 0.92 between domains. In addition, the SOSG-OQ 2.0 presented an excellent correlation with the SF-36. The construct consists of 27 (20 preoperative and 7 postoperative) questions. The preoperative questions constitute five domains: physical function (6 questions); neurological function (4 questions); pain (5 questions); mental function (2 questions); and social function (3 questions). All questions contain 5 items with scores ranging from 1 to 5. To obtain the total score on the SOSG-OQ, one needs to reverse the score on the items, that is, 1 = 5, 2 = 4, and so on. The higher the score, the worse the quality of life. The score of the seven postoperative questions is a percentage of the maximum potential points (rule of three). 21

## Results

The translations of the SOSG-OQ 2.0 by the 3 translators presented a high degree of similarity for most questions. The translators kept all question titles and subtitles and their internal and external orders.

As for the translation of the questions per se, there was little discrepancy between the reviewers, with only two questions showing significant divergence between them ( Table 1 ). There were some disagreements in the translation of the alternatives ( Table 2 ). With these divergences resolved, we prepared the amalgamated translation.

**Table 1:** Discrepancies in the translation of questions

Original	Alternative 1	Alternative 2	Conciliation
Do you require assistance from others to travel outside of the home?	Você precisa de ajuda de outras pessoas para sair de casa?	Você necessita de auxílio dos outros para trabalhos fora do ambiente domiciliar?	Você precisa de ajuda de outras pessoas para sair de casa?
When I feel pain, it is awful, and I feel that it overwhelms me.	Quando eu sinto dor, é horrível e sinto que isso me oprime.	Quando eu sinto dor, é uma dor horrível e insuportável.	Quando eu sinto dor, é uma dor horrível e insuportável.

**Table 2:** Examples of discrepancies regarding the translations of answer items

Original	Alternative 1	Alternative 2	Conciliation
Somewhat	Pouco	Mais ou menos	Mais ou menos
A little bit	Muito pouco	Um pouco	Um pouco
Sometimes	As vezes	Algumas vezes	Algumas vezes
Often	Frequentemente	Geralmente	Frequentemente

## Back-translation and Final Version

Two sworn translators, with native proficiency in English, performed the BT of the amalgamated text. Both BTs were quite similar, and any differences were solved by consensus among the main author and the sworn translators ( Table 3 ).

**Table 3:** Examples of discrepancies and consensus between the two back-translations

Back-translation 1	Back-translation 2	Consensus
A little	little	A little
%word% Constantly	Constantly %word%	%word% Constantly
Moderate outdoor activities	Moderate activity outside of the home	Moderate outdoor activities

Since none of the questions or alternatives was “extremely changed” compared with the original questionnaire, the amalgamated translation was the final considered version of the questionnaire ( Table 4 ).

**Table 4:** Reconciled translation and final version

**I- Função Física**
1. Qual é o seu nível de atividade?
Sem limitação nas atividades – Atividade moderada ao ar livre – Mobilidade mínima em residência – Restrito a deslocamento da cadeira para a cama – Acamado
2. Qual é a sua capacidade de trabalhar e/ou estudar?
Ilimitada – 4-8 horas por dia – 2-4 horas por dia – Menos de 2 horas por dia – Nenhuma
3. A sua coluna limita a sua habilidade de cuidar de si mesmo?
Não me atrapalha – Um pouco – Mais ou menos – Moderadamente - Bastante
4. Você precisa de assistência de outros para sair de casa?
Nunca – Raramente – Algumas vezes – Frequentemente – Muito frequentemente
5- Você precisa de assistência para caminhar?
Nenhuma – Bengala – Um andador ou duas bengalas – Auxílio de outras pessoas – Não posso caminhar
6. Você sai de casa para eventos sociais/socializar?
Nunca – Raramente – Algumas vezes – Frequentemente – Muito frequentemente
**IIA- Função neurológica dos membros inferiores**
7. Você sente fraqueza nas pernas?Nunca – Leve, ocasionalmente – Leve, constantemente – Moderada, constantemente – Severa, constantemente
**IIB- Função neurológica dos braços**
8. Você sente fraqueza nos braços?
Nunca – Leve, ocasionalmente – Leve, constantemente – Moderada, constantemente – Severa, constantemente
**IIC- Função neurológica intestinal**
9. Você sente dificuldade de controlar seu intestino (exceto em casos de diarreias)
Nunca – Leve, ocasionalmente – Leve, constantemente – Moderada, constantemente – Severa, constantemente
**IID- Função neurológica da bexiga**
10. Você sente dificuldade de controlar sua vontade de urinar?
Nunca – Raramente – Algumas vezes – Frequentemente – Faço uso de sonda
**III- Dor**
11. Em geral, quanto de dor nas costas você tem?
Nenhuma – Muito fraca – Fraca – Moderada – Severa
12. Quando você está na sua posição mais confortável, você continua a sentir dores?
Nunca – Raramente – Algumas vezes – Frequentemente – Muito frequentemente
13. Em geral, a dor nas costas limita sua mobilidade (sentar-se, andar, levantar-se…)?
Nunca – Raramente – Algumas vezes – Frequentemente – Muito frequentemente
14. Quão confiante você se sente em controlar a sua dor?
Não confio – Confio pouco – Confio moderadamente – Confio muito – Confio completamente
15. Quando eu sinto dor, é uma dor horrível e insuportável.
Nunca – Raramente – Algumas vezes – Frequentemente – Muito frequentemente
**IV- Saúde mental**
16. Você se sente deprimido?
Nunca – Raramente – Algumas vezes – Frequentemente – Muito frequentemente
17. Você sente ansiedade em relação ao seu estado de saúde?
Nunca – Raramente – Algumas vezes – Frequentemente – Muito frequentemente
**V- Função social**
18. Sua doença na coluna influencia na sua habilidade de concentração em conversas, leituras e ver televisão?
Nunca – Raramente – Algumas vezes – Frequentemente – Muito frequentemente
19. Você acha que sua doença na coluna atrapalha suas relações interpessoais?
Nunca – Raramente – Algumas vezes – Frequentemente – Muito frequentemente
20. Você se sente confortável em conhecer novas pessoas?
Nunca – Raramente – Algumas vezes – Frequentemente – Muito frequentemente
**Questões pós-operatórias**
21. Você está satisfeito com os resultados da sua cirurgia de remoção de tumor?
Muito satisfeito – Satisfeito – Nem satisfeito nem insatisfeito – Pouco insatisfeito – Muito insatisfeito
22. Se você pudesse escolher, faria o mesmo tratamento novamente?
Definitivamente sim – Provavelmente sim – Não sei dizer – Provavelmente não – Definitivamente não
23. Como sua cirurgia modificou sua função física e capacidade de realizar atividades do dia a dia?
Melhorou muito – Melhorou – Não mudou – Piorou um pouco – Piorou muito
24. Como sua cirurgia da coluna afetou sua medula e/ou raízes nervosas?
Melhorou muito – Melhorou – Não mudou – Piorou um pouco – Piorou muito
25. Como sua cirurgia afetou a sua dor na coluna?
Melhorou muito – Melhorou – Não mudou – Piorou um pouco – Piorou muito

## Discussion

Metastatic spinal tumors can cause different clinical manifestations and considerably impact the quality of life of the patients. This impact is not restricted to the affected spinal segment, due to the systemic characteristic of the disease. 4 28 29 In addition, the existing questionnaires to assess the clinical outcomes of patients with metastatic spinal tumors were nonspecific and did not involve all variables. 9 20 Thus, the SOSG-OQ 2.0 was developed in an attempt to quantify the impact of the condition on quality of life. 24 However, to date, no version of the questionnaire in Brazilian Portuguese had been published.

In the present study, we performed the translation into Brazilian Portuguese and cross-cultural adaptation of the SOSG-OQ 2.0. Despite some discrepancies among the initial translations, mainly regarding adverbs of degree ( *very* , *enough* , *little* etc.) reaching consensus among translators was simple. Likewise, the cross-cultural adaptation required few changes (such as altering *use of chopsticks* to *use of cutlery* ), since it was originally developed by American and European researchers, whose habits tend to be very similar to those of Brazilians. Similarly, in a study by the Brazilian Spine Study Group and Brazilian surgeons the Frailty Index 30 was translated; despite discrepancies regarding some items, few cross-cultural adaptations were required. 31

The SOSG-OQ consists of 27 questions, including 20 on the symptoms and impact of the disease on the patient's quality of life, plus 7 questions on how the patient feels about the surgical procedure. 20 21 In psychometric and consistency evaluation studies, 21 24 the SOSG-OQ correlated strongly with the quality-of-life scores on the EQ-5D and SF-36. In addition, its subgroups presented strong internal consistency. 21 24

The potential improvement in the follow-up and evaluation of the impact of spinal neoplasms using the SOSG-OQ has led several authors to translate it into their native languages. Luksanapruksa et al. 32 performed the translation and cross-cultural adaptation into Thai, and they reported that the domains pf the translated version maintained a high internal consistency (Cronbach alpha > 0.7) and that the questionnaire presented a strong correlation with the 5-Level EQ-5D (EQ-5D-5L). Likewise, Brodano et al. 33 reported the validity of the Italian version in terms of the internal domains and their correlation with the SF-36 subdomains, as well as a high consistency among questionnaire items.

Yin et al. 34 showed that the simplified Chinese version presented a strong correlation with the EQ-5D-5L and SF-36, an excellent internal consistency among its subgroups, and good intra-observer results. A group of researchers 35 recently demonstrated that the physical function, pain interference, and depression domains of the PROMIS presented a strong correlation with the SOSG-OQ.

Regarding the impact of the SOSG-OQ on decision-making, a 2020 study 36 on the benefits of potentially predicting scores on health-related quality of life (HRQoL) instruments after the surgical treatment of spinal neoplasms pointed out that the 2 questionnaires most beneficial in terms of the prediction of postoperative outcomes were the EQ-5D and the SOSG-OQ. 36 Furthermore, an article published in 2021 37 proposed the development of a summarized version of the SOSG-OQ especially focused on utility units, which would enable its use in the analysis of decisions, such as the one to convert these utility units into quality-adjusted life years (QALYs).

The present study has limitations, mainly the non-validation of the Brazilian Portuguese version due to the difficulties in obtaining sufficient data. However, it will serve as a basis for future validation. We believe that validation studies of our version of the SOSG-OQ are required for the internal consistency of its constructs and to determine its correlation with already established questionnaires, such as the EQ-5D.

## Conclusion

In the present study, we performed the cross-cultural adaptation and translation into Brazilian Portuguese of the SOSG-OQ, which presents semantic validity regarding the original English version, which enables its application to the Brazilian population, adding another tool for spine surgeons to monitor this complex group of patients.

## References

[1] Murray C JL Vos T Lozano R Disability-adjusted life years (DALYs) for 291 diseases and injuries in 21 regions, 1990-2010: a systematic analysis for the Global Burden of Disease Study 2010 Lancet 2012 380 9859 2197 2223 23245608 10.1016/S0140-6736(12)61689-4

[2] Wai E K Finkelstein J A Tangente R P Quality of life in surgical treatment of metastatic spine disease Spine 2003 28 05 508 512 12616166 10.1097/01.BRS.0000048646.26222.FA

[3] Boing A F Vargas S AL Boing A C [The burden of neoplasm in Brazil: mortality and hospital morbidity from 2002 to 2004] Rev Assoc Med Bras 2007 53 04 317 322 17823734 10.1590/s0104-42302007000400016

[4] Choi D Bilsky M Fehlings M Fisher C Gokaslan Z Spine Oncology-Metastatic Spine Tumors Neurosurgery 2017 80(3S):S131 S137 28350950 10.1093/neuros/nyw084

[5] Morgen S S Engelholm S A Larsen C F Søgaard R Dahl B Health-related quality of life in patients with metastatic spinal cord compression Orthop Surg 2016 8 03 309 315 27627713 10.1111/os.12253 PMC6584430

[6] Barzilai O Fisher C G Bilsky M H State of the art treatment of spinal metastatic disease Neurosurgery 2018 82 06 757 769 29481645 10.1093/neuros/nyx567

[7] Barzilai O McLaughlin L Lis E Yamada Y Bilsky M H Laufer I Outcome analysis of surgery for symptomatic spinal metastases in long-term cancer survivors J Neurosurg Spine 2019 31 02 1 6 31026814 10.3171/2019.2.SPINE181306

[8] Barcena A Lobato R D Rivas J J Spinal metastatic disease: analysis of factors determining functional prognosis and the choice of treatment Neurosurgery 1984 15 06 820 827 6514154

[9] Street J Berven S Fisher C Ryken T Health related quality of life assessment in metastatic disease of the spine: a systematic review Spine 2009 34(22, Suppl)S128 S134 19829272 10.1097/BRS.0b013e3181b778b2

[10] Frankel H L Hancock D O Hyslop G The value of postural reduction in the initial management of closed injuries of the spine with paraplegia and tetraplegia. I Paraplegia 1969 7 03 179 192 5360915 10.1038/sc.1969.30

[11] El Masry W S Tsubo M Katoh S El Miligui Y HS Khan A Validation of the American Spinal Injury Association (ASIA) motor score and the National Acute Spinal Cord Injury Study (NASCIS) motor score Spine 1996 21 05 614 619 8852318 10.1097/00007632-199603010-00015

[12] Karimi M Brazier J Health, Health-Related Quality of Life, and Quality of Life: What is the Difference? PharmacoEconomics 2016 34 07 645 649 26892973 10.1007/s40273-016-0389-9

[13] LIVSFORSK network Haraldstad K Wahl A Andenæs R A systematic review of quality of life research in medicine and health sciences Qual Life Res 2019 28 10 2641 2650 31187410 10.1007/s11136-019-02214-9 PMC6761255

[14] Fairbank J CT Pynsent P B The oswestry disability index Spine 2000 25 22 2940 2952, discussion 2952 11074683 10.1097/00007632-200011150-00017

[15] Cook C Richardson J K Braga L Cross-cultural adaptation and validation of the Brazilian Portuguese version of the Neck Disability Index and Neck Pain and Disability Scale Spine 2006 31 14 1621 1627 16778699 10.1097/01.brs.0000221989.53069.16

[16] McHorney C A Ware J E Jr Lu J F Sherbourne C D The MOS 36-item Short-Form Health Survey (SF-36): III. Tests of data quality, scaling assumptions, and reliability across diverse patient groups Med Care 1994 32 01 40 66 8277801 10.1097/00005650-199401000-00004

[17] Rabin R de Charro F EQ-5D: a measure of health status from the EuroQol Group Ann Med 2001 33 05 337 343 11491192 10.3109/07853890109002087

[18] Young J Badgery-Parker T Dobbins T Comparison of ECOG/WHO performance status and ASA score as a measure of functional status J Pain Symptom Manage 2015 49 02 258 264 24996034 10.1016/j.jpainsymman.2014.06.006

[19] Choi D Morris S Crockard A Assessment of quality of life after surgery for spinal metastases: position statement of the Global Spine Tumour Study Group World Neurosurg 2013 80 06 e175 e179 23422266 10.1016/j.wneu.2013.02.054

[20] Street J Lenehan B Berven S Fisher C Introducing a new health-related quality of life outcome tool for metastatic disease of the spine: content validation using the International Classification of Functioning, Disability, and Health; on behalf of the Spine Oncology Study Group Spine 2010 35 14 1377 1386 20505561 10.1097/BRS.0b013e3181db96a5

[21] Janssen S J Teunis T van Dijk E Validation of the Spine Oncology Study Group-Outcomes Questionnaire to assess quality of life in patients with metastatic spine disease Spine J 2017 17 06 768 776 26254565 10.1016/j.spinee.2015.07.456

[22] PROMIS Cooperative Group Cella D Yount S Rothrock N The Patient-Reported Outcomes Measurement Information System (PROMIS): progress of an NIH Roadmap cooperative group during its first two years Med Care 2007 45(5, Suppl 1)S3 S11 10.1097/01.mlr.0000258615.42478.55 PMC2829758 17443116

[23] Paulino Pereira N R Janssen S J Raskin K A Most efficient questionnaires to measure quality of life, physical function, and pain in patients with metastatic spine disease: a cross-sectional prospective survey study Spine J 2017 17 07 953 961 28242336 10.1016/j.spinee.2017.02.006

[24] AOSpine Knowledge Forum Tumor Versteeg A L Sahgal A Rhines L D Psychometric evaluation and adaptation of the Spine Oncology Study Group Outcomes Questionnaire to evaluate health-related quality of life in patients with spinal metastases Cancer 2018 124 08 1828 1838 29409108 10.1002/cncr.31240 PMC5900572

[25] Beaton D E Bombardier C Guillemin F Ferraz M B Guidelines for the process of cross-cultural adaptation of self-report measures Spine 2000 25 24 3186 3191 11124735 10.1097/00007632-200012150-00014

[26] Reichenheim M E Moraes C L Operacionalização de adaptação transcultural de instrumentos de aferição usados em epidemiologia Rev Saude Publica 2007 41 04 665 673 17589768 10.1590/s0034-89102006005000035

[27] Coster W J Mancini M C Recommendations for translation and cross-cultural adaptation of instruments for occupational therapy research and practice Rev Ter Ocup Univ Sao Paulo 2015 26 01 50 57

[28] White A P Kwon B K Lindskog D M Friedlaender G E Grauer J N Metastatic disease of the spine J Am Acad Orthop Surg 2006 14 11 587 598 17030592 10.5435/00124635-200610000-00001

[29] Gerszten P C Spine metastases: from radiotherapy, surgery, to radiosurgery Neurosurgery 2014 61 01 16 25 25032525 10.1227/NEU.0000000000000375

[30] European Spine Study Group International Spine Study Group Miller E K Vila-Casademunt A Neuman B J External validation of the adult spinal deformity (ASD) frailty index (ASD-FI) Eur Spine J 2018 27 09 2331 2338 29603013 10.1007/s00586-018-5575-3

[31] Pratali R R Romerio C FWE Daher M T Adaptation of the frailty index for brazilian portuguese in adult spine deformity surgery Coluna/Columna 2020 19 03 168 171

[32] Luksanapruksa P Phikunsri P Trathitephun W Validity and reliability of the Thai version of the Spine Oncology Study Group Outcomes Questionnaire version 2.0 to assess Quality of Life in Patients with Spinal Metastasis Spine J 2021 21 11 1920 1924 34010685 10.1016/j.spinee.2021.05.010

[33] Brodano G B Pesce E Griffoni C Adaptation and Validation of the Spine Oncology Study Group Outcomes Questionnaire in Italian LanguageGlobal Spine J 2022;2022(00):21925682221083913 10.1177/21925682221083913 PMC10538325 35344384

[34] Yin M Sun Z Ding X Cross-cultural adaptation and validation of simplified Chinese version of the Spine Oncology Study Group Outcomes Questionnaire (SOSGOQ) 2.0 with its assessment in clinical setting Spine J 2022 22 12 2024 2032 36031097 10.1016/j.spinee.2022.08.013

[35] Richardson M A Bernstein D N Kulp A Mesfin A Patient Reported Outcomes in Metastatic Spine Disease: Concurrent Validity of PROMIS with the Spine Oncology Study Group Outcome Questionnaire Spine 2022 47 08 591 596 35102119 10.1097/BRS.0000000000004327

[36] Feghali J Pennington Z Ehresman J Predicting postoperative quality-of-life outcomes in patients with metastatic spine disease: who benefits? J Neurosurg Spine 2020 34 03 1 7 33338994 10.3171/2020.7.SPINE201136

[37] AO Spine Knowledge Forum Tumor Pahuta M A Fisk F Versteeg A L Calculating Utilities From the Spine Oncology Study Group Outcomes Questionnaire: A Necessity for Economic and Decision Analysis Spine 2021 46 17 1165 1171 34334684 10.1097/BRS.0000000000003981 PMC8357033

