# Supplementary material for: Translation and Cross-cultural Adaptation of the SOSG-OQ 2.0 Questionnaire into Brazilian Portuguese
Source: Rev Bras Ortop (Sao Paulo). 2024 Mar 21;59(1):e38–45. doi: 10.1055/s-0043-1775890 (PMC10957269; doi:10.1055/s-0043-1775890)
Supplement: Related texts

# Research article: Tradução e adaptação transcultural do questionário SOSG-OQ 2.0 para o português brasileiro *

**Keywords:** coluna vertebral/cirurgia, pesquisas e questionários, metástase neoplásica, qualidade de vida, traduções

## Abstract

**Objetivo:** Realizar a adaptação transcultural e a tradução para o português brasileiro da versão 2.0 do Questionário de Desfechos do Spine Oncology Study Group (Spine Oncology Study Group – Outcomes Questionnaire 2.0, SOSG-OQ 2.0, em inglês) para viabilizar sua aplicação em pacientes brasileiros e permitir a utilização deste questionário que está em voga na literatura científica por pesquisadores brasileiros.

**Materiais e Métodos:** Trata-se de uma pesquisa básica, não randomizada, não comparativa. As etapas de tradução foram realizadas conforme propostas por Reichenheime e Moraes, principalmente as sessões de equivalência semântica e equivalência de mensuração, e também foram seguidas as recomendações de Coster e Mancini, principalmente na etapa de tradução. As etapas foram as seguintes: primeira – tradução do questionário para o português brasileiro; segunda – retroversão; terceira – comparação semântica; e quarta – validação final do constructo.

**Resultados:** As traduções do SOSG-OQ 2.0 feitas por três tradutores apresentaram grande similaridade na maioria das questões. Todos os títulos e subtítulos de perguntas foram mantidos pelos tradutores, assim como as ordens interna e externa das perguntas. A retroversão da tradução conciliada foi realizada por dois tradutores juramentados, com fluência nativa na língua inglesa. Ambas as retroversões foram bastante similares, as divergências foram sanadas por consenso entre o autor principal e os tradutores juramentados, e a versão traduzida foi considerada a versão final.

**Conclusão:** Neste estudo, apresenta-se uma versão traduzida do SOSG-OQ 2.0 que tem validade semântica com a versão original publicada em inglês, o que permite a sua aplicação na população brasileira, e acrescenta mais uma ferramenta para que os cirurgiões de coluna possam acompanhar de forma melhor este complexo grupo de pacientes.

## Introdução

No Brasil, desde o ano 2000, o câncer é a segunda maior causa de mortalidade, atrás apenas das doenças cardíacas. 1 Além disso, os tumores metastáticos da coluna vertebral têm alta prevalência se comparada à dos tumores primários da coluna vertebral. 2 3 A doença metastática da coluna vertebral é responsável pelo aumento da morbidade relacionada à patologia primária, e tem impacto direto na qualidade de vida do paciente. 2 4 5

Não é incomum que os pacientes acometidos pela doença metastática apresentem disfunções que envolvem vários sistemas do organismo humano, e eles podem ser submetidos a diversos tratamentos, como quimioterapia e/ou radioterapia. 6 A cirurgia da coluna vertebral às vezes requer procedimentos para preservar ou recuperar a função neurológica, manuter a estabilidade segmentar vertebral e controlar de dor. 6 7 8

Atualmente há múltiplas ferramentas disponíveis para se estudar os desfechos clínicos de pacientes com tumor metastático da coluna; elas são inespecíficas e, em grande parte, utilizadas para a análise de uma única variável, 9 como as avaliações protocoladas pela escala de Frankel ou pela Escala de Deficiência da American Spinal Injury Association (ASIA), por exemplo, que objetivam quantificar (classificar) o grau de lesão neurológica do paciente. 10 11 Adicionalmente, existem os questionários de qualidade de vida preenchidos pelos pacientes, que buscam quantificar como o paciente avalia sua qualidade de vida/estado de saúde, e permitem a identificação do impacto de um procedimento ou patologia. 12 13 Os questionários mais utilizados na prática da cirurgia da coluna vertebral são o Índice de Incapacidade de Oswestry 14 e o Índice de Incapacidade Cervical, 15 que foram desenvolvidos especificamente para quantificar o impacto da patologia, nas regiões da coluna lombar e da coluna cervical, respectivamente, sobre a qualidade de vida percebida pleos próprios pacientes. Ademais, existem questionários mais amplos, como o de Cinco Dimensões do EuroQoL (EuroQoL Five Dimensions, EQ-5D) ou a Pesquisa de Formulário Curto de 36 Itens (36-Item Short Form Survey, SF-36), cujo objetivo é quantificar a qualidade de vida dos pacientes de maneira mais abrangente, sem se ater a uma patologia ou região específicas, o que permite a comparação de pacientes com diferentes históricos de doença ou tratamento em uma mesma escala. 16 17 Por fim, existem também as escalas de impacto na qualidade de vida, que são avaliadas pelos médicos e/ou cirurgião responsável pelo caso, como a escala do Eastern Cooperative Oncology Group (ECOG), que visa classificar o impacto do tumor nas atividades do paciente, sendo o valor 0 igual à qualidade de vida normal, e 5, equivalente à morte. 18

Apesar de validados e úteis para o acompanhamento e avaliação de pacientes com tumores metastáticos, nenhum desses questionários se centra especificamente em pacientes com tumor de coluna vertebral, o que faz com que a literatura apresente divergências sobre qual seria a melhor combinação de questionários para realizar o acompanhamento de pacientes com tumores metastáticos da coluna. Street et al., 9 por exemplo, recomendam o uso da escala do ECOG e do SF-36, ao passo que Choi et al. 19 recomendam a utilização do EQ-5D.

Em decorrência da ausência de questionários que objetivam a avaliação específica para do tumor metastático da coluna vertebral, o Spine Oncologic Study Group (SOSG) desenvolveu um questionário de desfechos (SOSG – Outcomes Questionnaire, SOG-OQ) para análise de qualidade de vida dos pacientes portadores dessa patologia. 20 Ademais, um estudo 21 demonstrou que o SOSG-OQ é superior à versão de 3 níveis do EQ-5D (3-Level Version, EQ-5D-3L) em pacientes com metástase, linfoma ou mieloma. Além disso, o SOSG-OQ se mostrou mais eficaz do que o Sistema de Informações de Medidas de Desfecho Relatadas pelos Pacientes (Patient-Reported Outcomes Measurement Information System, PROMIS) 22 para analisar a qualidade de vida de pacientes com metástase de coluna, apesar de o PROMIS se apresentar mais eficaz na avaliação de função física e dor, segundo o estudo de Paulino Pereira et al. 23

Apesar de pensado para abranger especificamente pacientes com metástases na coluna, o SOSG-OQ ainda tinha algumas inconsistências internas que faziam com que os itens de alguns de seus subdomínios não apresentassem correlação muito eficaz. 21 24

Deste modo, em 2018, Veersteg et al. 24 realizaram um estudo psicométrico sobre o SOSG-OQ, no qual foi desenvolvida uma versão atualizada do questionário. Para solucionar as discrepâncias da primeira versão, os autores dividiram a questão 8 do formulário original (sobre a função do intestino e da bexiga), em 2 questões distintas, para facilitar o preenchimento, tendo em vista que, muitas vezes, apenas um dos órgãos, o intestino ou a bexiga, apresentam disfunção. Ademais, as questões 7 (sobre auxílio para caminhar) e 20 (sobre sair da casa) foram transferidas para o domínio de função física, e a questão 16 foi movida para o domínio de dor, ao passo que a questão 15 (sobre o nível de energia) foi excluída, por não apresentar associação com nenhum domínio e não render informação significativa o suficiente para o resultado. 24 Por fim, a segunda versão do questionário, o constructo SOSG-OQ 2.0, foi comparada com a Escala Numérica de Avaliação (Numeric Rating Scale, NRS) da dor e com SF-36 em pacientes com metástase da coluna vertebral, e demonstrou-se forte correlação entre os questionários. 24

Deste modo, o objetivo deste trabalho foi realizar a adaptação transcultural e a tradução para o português brasileiro do SOSG-OQ 2.0, para viabilizar a aplicação do questionário em pacientes brasileiros.

## Materiais e Métodos

Pesquisa básica, não randomizada, não comparativa.

## Processo de tradução e adaptação transcultural

O processo de tradução e adaptação transcultural de um instrumento envolve múltiplas etapas para garantir que o constructo traduzido seja não só válido e equivalente como também faça sentido para o público-alvo. 25 O processo se inicia com a realização múltiplas traduções do questionário original, que são então sintetizadas para que se obtenha uma versão traduzida conciliada. Após o consenso na tradução, um grupo de tradutores (com fluência nativa na língua original) faz retroversões (RVs) do documento, que são sintetizadas para se obter a RV final. Então, a RV é comparada à versão original por um comitê de especialistas, que irá verificar se há discrepâncias entre as duas; caso haja poucas ou nenhuma discrepância, o constructo pode ser testado na população-alvo para avaliações psicométricas e de validade. 25 26

As etapas de tradução foram realizadas conforme proposto por Reichenheime e Moraes, 26 principalmente as sessões de equivalência semântica e equivalência de mensuração, e também seguiram as recomendações feitas por Coster e Mancini, 27 principalmente na etapa de tradução ( Fig. 1 ).

**Fig. 1:** Diagrama de fluxo da tradução do SOSG-OQ até obtenção da versão final.

**Passo 1:** tradução do questionário SOSG-OQ 2.0 para o português brasileiro, feita individualmente por 3 pesquisadores brasileiros. As três traduções foram comparadas para se elaborar uma versão unificada por consenso entre os pesquisadores, chamada de tradução conciliada. Durante a etapa de tradução, pediu-se aos revisores que, caso julgassem necessário, realizassem alterações em elementos das perguntas que não fossem muito familiares para a população brasileira.

**Passo 2:** a tradução conciliada foi enviada para dois revisores, especialistas em letras certificados com proficiência na língua inglesa. A conciliação destes questionários foi chamada de RV conciliada.

**Passo 3 e 4:** Por fim, um outro tradutor (chamado de tradutor final), que não esteve envolvido em nenhuma das traduções e RVs, comparou a RV conciliada e a versão original para dar seu parecer sobre a similaridade dos questionários em aspectos denotativos e conotativos, e podia avaliar as questões como *inalteradas* , *pouco alteradas* , ou *muito alteradas* .

## SOSG-OQ 2.0

Elaborado em 2018 como uma adaptação do original, o SOSG-OQ 2.0, tem o intuito de melhorar a validade interna de seus domínios e a sua correlação com outros constructos já validados. 24 Os valores de confiabilidade do questionário nas avaliações de tese-reteste variaram de 0,58 a 0,92 entre os domínios, e o instrumento apresentou excelente correlação com o SF-36. O constructo é composto por 27 questões, 20 pré-operatórias e 7 pós-operatórias, e as pré-operatórias estão divididas em 5 domínios: função física (6 questões); função neurológica (4 questões); dor (5 questões); funções mentais (2 questões); e funções sociais (3 questões). Todas as questões têm 5 itens com pontuação de 1 a 5 e, para se obter a pontuação total do SOSG-OQ, é preciso inverter a pontuação dos itens, ou seja, 1 = 5, 2 = 4, e assim por diante. Quanto maior a pontuação, pior a qualidade de vida do paciente. Para as sete questões pós-operatórias, a pontuação do questionário se dá por uma porcentagem dos pontos máximos possíveis (regra de três). 21

## Resultados

As traduções do SOSG-OQ feitas pelos três tradutores apresentaram grande similaridade na maioria das questões. Todos os títulos e subtítulos de perguntas foram mantidos, assim como as ordens interna e externa das perguntas.

Quanto à tradução das perguntas propriamente ditas, houve pouca discrepância entre os revisores, e só houve divergências significativas com relação a duas perguntas ( Tabela 1 ). Ademais, houve discordâncias também na elaboração da tradução das alternativas ( Tabela 2 ). Sanadas as divergências, a tradução conciliada foi elaborada.

**Tabela 1:** Discrepâncias ocorridas na tradução das perguntas

Original	Alternativa 1	Alternativa 2	Conciliação
Do you require assistance from others to travel outside of the home?	Você precisa de ajuda de outras pessoas para sair de casa?	Você necessita de auxílio dos outros para trabalhos fora do ambiente domiciliar?	Você precisa de ajuda de outras pessoas para sair de casa?
When I feel pain, it is awful, and I feel it overwhelms me.	Quando eu sinto dor, é horrível e sinto que isso me oprime.	Quando eu sinto dor, é uma dor horrível e insuportável.	Quando eu sinto dor, é uma dor horrível e insuportável.

**Tabela 2:** Exemplos de discrepâncias entre as traduções nos itens de respostas

Original	Alternativa 1	Alternativa 2	Conciliação
Somewhat	Pouco	Mais ou menos	Mais ou menos
A little bit	Muito pouco	Um pouco	Um pouco
Sometimes	Às vezes	Algumas vezes	Algumas vezes
Often	Frequentemente	Geralmente	Frequentemente

## Retroversão e Obtenção de versão final

As RVs foram feitas por dois tradutores juramentados com fluência nativa na língua inglesa. Ambas foram bastante similares, e as divergências foram sanadas por consenso entre o autor principal e os tradutores juramentados ( Tabela 3 ).

**Tabela 3:** Exemplos de discrepâncias e consenso entre as duas retroversões

Retroversão 1	Retroversão 2	Consenso
A little	little	A little
%palavra%Constantly	Constantly %palavra%	%palavra%Constantly
Moderate outdoor activities	Moderate activity outside of the home	Moderate outdoor activities

Ademais, nenhuma das questões ou alternativas apresentou-se como “muito alterada” em comparação com a versão original do questionário, de modo que a tradução conciliada foi considerada como a versão final do questionário ( Tabela 4 ).

**Tabela 4:** Tradução conciliada e versão final

**I- Função Física**
1. Qual é o seu nível de atividade?
Sem limitação nas atividades – Atividade moderada ao ar livre – Mobilidade mínima em residência – Restrito a deslocamento da cadeira para a cama – Acamado
2. Qual é a sua capacidade de trabalhar e/ou estudar?
Ilimitada – 4-8 horas por dia – 2-4 horas por dia – Menos de 2 horas por dia – Nenhuma
3. A sua coluna limita a sua habilidade de cuidar de si mesmo?
Não me atrapalha – Um pouco – Mais ou menos – Moderadamente - Bastante
4. Você precisa de assistência de outros para sair de casa?
Nunca – Raramente – Algumas vezes – Frequentemente – Muito frequentemente
5- Você precisa de assistência para caminhar?
Nenhuma – Bengala – Um andador ou duas bengalas – Auxílio de outras pessoas – Não posso caminhar
6. Você sai de casa para eventos sociais/socializar?
Nunca – Raramente – Algumas vezes – Frequentemente – Muito frequentemente
**IIA- Função neurológica dos membros inferiores**
7. Você sente fraqueza nas pernas?Nunca – Leve, ocasionalmente – Leve, constantemente – Moderada, constantemente – Severa, constantemente
**IIB- Função neurológica dos braços**
8. Você sente fraqueza nos braços?
Nunca – Leve, ocasionalmente – Leve, constantemente – Moderada, constantemente – Severa, constantemente
**IIC- Função neurológica intestinal**
9. Você sente dificuldade de controlar seu intestino (exceto em casos de diarreias)
Nunca – Leve, ocasionalmente – Leve, constantemente – Moderada, constantemente – Severa, constantemente
**IID- Função neurológica da bexiga**
10. Você sente dificuldade de controlar sua vontade de urinar?
Nunca – Raramente – Algumas vezes – Frequentemente – Faço uso de sonda
**III- Dor**
11. Em geral, quanto de dor nas costas você tem?
Nenhuma – Muito fraca – Fraca – Moderada – Severa
12. Quando você está na sua posição mais confortável, você continua a sentir dores?
Nunca – Raramente – Algumas vezes – Frequentemente – Muito frequentemente
13. Em geral, a dor nas costas limita sua mobilidade (sentar-se, andar, levantar-se…)?
Nunca – Raramente – Algumas vezes – Frequentemente – Muito frequentemente
14. Quão confiante você se sente em controlar a sua dor?
Não confio – Confio pouco – Confio moderadamente – Confio muito – Confio completamente
15. Quando eu sinto dor, é uma dor horrível e insuportável.
Nunca – Raramente – Algumas vezes – Frequentemente – Muito frequentemente
**IV- Saúde mental**
16. Você se sente deprimido?
Nunca – Raramente – Algumas vezes – Frequentemente – Muito frequentemente
17. Você sente ansiedade em relação ao seu estado de saúde?
Nunca – Raramente – Algumas vezes – Frequentemente – Muito frequentemente
**V- Função social**
18. Sua doença na coluna influencia na sua habilidade de concentração em conversas, leituras e ver televisão?
Nunca – Raramente – Algumas vezes – Frequentemente – Muito frequentemente
19. Você acha que sua doença na coluna atrapalha suas relações interpessoais?
Nunca – Raramente – Algumas vezes – Frequentemente – Muito frequentemente
20. Você se sente confortável em conhecer novas pessoas?
Nunca – Raramente – Algumas vezes – Frequentemente – Muito frequentemente
**Questões pós-operatórias**
21. Você está satisfeito com os resultados da sua cirurgia de remoção de tumor?
Muito satisfeito – Satisfeito – Nem satisfeito nem insatisfeito – Pouco insatisfeito – Muito insatisfeito
22. Se você pudesse escolher, faria o mesmo tratamento novamente?
Definitivamente sim – Provavelmente sim – Não sei dizer – Provavelmente não – Definitivamente não
23. Como sua cirurgia modificou sua função física e capacidade de realizar atividades do dia a dia?
Melhorou muito – Melhorou – Não mudou – Piorou um pouco – Piorou muito
24. Como sua cirurgia da coluna afetou sua medula e/ou raízes nervosas?
Melhorou muito – Melhorou – Não mudou – Piorou um pouco – Piorou muito
25. Como sua cirurgia afetou a sua dor na coluna?
Melhorou muito – Melhorou – Não mudou – Piorou um pouco – Piorou muito

## Discussão

Os tumores metastáticos da coluna vertebral podem gerar manifestações clínicas diversas e impactar consideravelmente a qualidade de vida dos pacientes, que não se restringem somente ao segmento da coluna acometido, devido à sua característica sistêmica. 4 28 29 Além disso, os questionários existentes para estudo dos desfechos clínicos de pacientes com tumor metastático da coluna vertebral eram inespecíficos e não envolviam todas as variáveis. 9 20 Deste modo, o SOSG-OQ 2.0 foi elaborado para tentar quantificar o impacto dessa doença na qualidade de vida dos pacientes. 24 No entanto, até agora, nenhuma versão do questionário em português brasileiro havia sido publicada.

No presente estudo, realizou-se a tradução para o português brasileiro e a adaptação transcultural do SOSG-OQ 2.0. Apesar de ter havido algumas discrepâncias entre as traduções iniciais, principalmente com relação aos advérbios de intensidade ( *muito* , *bastante* , *pouco* etc.) a obtenção de consenso entre os tradutores foi simples. Do mesmo modo, quanto à adaptação transcultural, quase nenhuma alteração foi necessária (como trocar *uso de hashis* por *uso de talheres* , por exemplo), tendo em vista que o questionário original foi elaborado em conjunto por pesquisadores americanos e europeus, cujos hábitos, de maneira mais generalizada, são bem similares aos dos brasileiros. De maneira similar, em trabalho realizado pelo Brazilian Spine Study Group em conjunto com outros cirurgiões brasileiros, traduziu-se o Índice de Fragilidade (Frailty Index), 30 e, apesar da ocorrência discrepâncias entre alguns itens, poucas adaptações culturais foram necessárias. 31

O SOSG-OQ é composto por 27 questões, sendo 20 baseadas nos sintomas e impactos da doença na qualidade de vida, e sete questões sobre como o paciente se sente em relação ao procedimento cirúrgico. 20 21 Em estudos de avaliação psicométrica e de consistência, 21 24 o SOSG-OQ se mostrou fortemente correlacionado com a pontuação de qualidade de vida obtida no EQ-5D e no SF-36, e os seus subgrupos apresentaram forte consistência interna.

Devido ao possível benefício de melhorar o acompanhamento e a avaliação do impacto das neoplasias na coluna fornecido pelo SOSG-OQ, diversos autores passaram a traduzi-lo para suas línguas nativas. Luksanapruksa et al. 32 realizaram a tradução e adaptação transcultural para o tailandês, e demonstraram que os domínios da versão traduzida mantiveram uma alta consistência interna (alpha de Cronbach > 0.7), e que o questionário manteve forte correlação com as pontuações obtidas na versão de 5 níveis do EQ-5D (EQ-5D-5L). Da mesma forma, Brodano et al., 33 demostraram a validade da versão italiana tanto em relação aos domínios internos quanto à sua correlação com os subdomínios do SF-36, além da alta consistência entre os itens do questionário.

Yin et al. 34 demonstraram que a versão chinesa simplificada demonstrou forte correlação tanto com o EQ-5D-5L quanto com o SF-36, além de apresentar excelente consistência interna entre seus subgrupos e bom resultado intraobservador. Por fim, um grupo de pesquisadores 35 demonstrou recentemente que os domínios de função física, de interferência da dor e de depressão PROMIS tinham forte correlação com as pontuações obtidas no SOSG-OQ.

Em relação ao impacto do SOSG-OQ na tomada de decisões, um estudo de 2020, 36 sobre os benefícios da possibilidade de previsão de pontuações em instrumentos de avaliação de qualidade de vida relativa à saúde (QVRS) no pós-operatório de cirurgias de neoplasias da coluna, apontou que os 2 questionários cuja capacidade de previsão de valores pós-operatórios poderiam trazer mais benefícios seriam o EQ-5D e o SOSG-OQ. Além disso, em artigo publicado em 2021, 37 foi proposta uma maneira de se fazer uma versão resumida do SOSG-OQ, especialmente centrada na obtenção das unidades utilitárias, o que permitiria a sua utilização em análises de decisões, como a de converter essas unidades utilitárias em anos de vida ajustados pela qualidade (AVAQ).

Este estudo apresenta limitações, sendo a principal a não validação da versão para o português brasileiro do instrumento, devido às dificuldades de se obter dados suficientes para realizá-la. No entanto, servirá como base para a realização de uma validação em um momento futuro, pois acreditamos que estudos de validação da versão em português brasileiro do SOSG-OQ são necessários para a consistência interna de seus constructos e a sua correlação com questionários já estabelecidos, como o EQ-5D.

## Conclusão

Este estudo apresenta uma versão traduzida para o português brasileiro do SOSG-OQ que apresenta validade semântica com a versão original em inglês, o que permite a sua aplicação na população brasileira, e acrescenta mais uma ferramenta para que os cirurgiões de coluna possam acompanhar este complexo grupo de pacientes.
